# Quinoline-2-carbonitrile–fumaric acid (1/0.5)

**DOI:** 10.1107/S1600536810032745

**Published:** 2010-08-21

**Authors:** Wan-Sin Loh, Ching Kheng Quah, Madhukar Hemamalini, Hoong-Kun Fun

**Affiliations:** a X-ray Crystallography Unit, School of Physics, Universiti Sains Malaysia, 11800 USM, Penang, Malaysia

## Abstract

The asymmetric unit of the title compound, C_10_H_6_N_2_·0.5C_4_H_4_O_4_, consists of one quinoline-2-carbonitrile mol­ecule and a half-mol­ecule of fumaric acid, which lies on an inversion center. The quinoline-2-carbonitrile mol­ecule is almost planar, with an r.m.s. deviation of 0.008 (1) Å. The acid and base are linked together *via* pairs of inter­molecular C—H⋯O and O—H⋯N hydrogen bonds, forming *R*_2_^2^(8) ring motifs. In the crystal, the carbonitrile mol­ecules are further linked by inter­molecular C—H⋯N hydrogen bonds, generating *R*_2_^2^(10) ring motifs, resulting in zigzag chains running along the *c* axis.

## Related literature

For the biological activity and syntheses of quinoline derivatives, see: Sasaki *et al.* (1998 ▶); Reux *et al.* (2009 ▶). For related structures, see: Loh, Fun *et al.* (2010 ▶); Loh, Quah *et al.* (2010 ▶); Quah *et al.* (2010 ▶). For hydrogen-bond motifs, see: Bernstein *et al.* (1995 ▶). For the stability of the temperature controller used in the data collection, see: Cosier & Glazer (1986 ▶). For reference bond-length data, see: Allen *et al.* (1987 ▶).
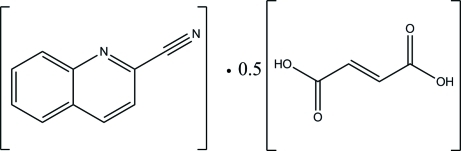


## Experimental

### 

#### Crystal data

C_10_H_6_N_2_·0.5C_4_H_4_O_4_
*M* *_r_* = 212.20
Monoclinic, 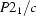

*a* = 3.7239 (1) Å
*b* = 19.1958 (3) Å
*c* = 13.6454 (2) Å
β = 93.805 (1)°
*V* = 973.27 (3) Å^3^
*Z* = 4
Mo *K*α radiation
μ = 0.10 mm^−1^
*T* = 100 K
0.17 × 0.15 × 0.09 mm

#### Data collection

Bruker SMART APEXII CCD area-detector diffractometer
Absorption correction: multi-scan (*SADABS*; Bruker, 2009 ▶) *T*_min_ = 0.983, *T*_max_ = 0.991
10682 measured reflections
2566 independent reflections
1983 reflections with *I* > 2σ(*I*)
*R*_int_ = 0.032

#### Refinement

*R*[*F* ^2^ > 2σ(*F* ^2^)] = 0.047
*wR*(*F* ^2^) = 0.128
*S* = 1.06
2566 reflections
149 parameters
H atoms treated by a mixture of independent and constrained refinement
Δρ_max_ = 0.37 e Å^−3^
Δρ_min_ = −0.26 e Å^−3^

### 

Data collection: *APEX2* (Bruker, 2009 ▶); cell refinement: *SAINT* (Bruker, 2009 ▶); data reduction: *SAINT*; program(s) used to solve structure: *SHELXTL* (Sheldrick, 2008 ▶); program(s) used to refine structure: *SHELXTL*; molecular graphics: *SHELXTL*; software used to prepare material for publication: *SHELXTL* and *PLATON* (Spek, 2009 ▶).

## Supplementary Material

Crystal structure: contains datablocks global, I. DOI: 10.1107/S1600536810032745/wn2403sup1.cif

Structure factors: contains datablocks I. DOI: 10.1107/S1600536810032745/wn2403Isup2.hkl

Additional supplementary materials:  crystallographic information; 3D view; checkCIF report

## Figures and Tables

**Table 1:** Hydrogen-bond geometry (Å, °)

*D*—H⋯*A*	*D*—H	H⋯*A*	*D*⋯*A*	*D*—H⋯*A*
O2—H1*O*2⋯N1	0.92 (2)	1.83 (2)	2.7272 (16)	167 (2)
C2—H2*A*⋯O1	0.93	2.44	3.3300 (19)	161
C8—H8*A*⋯N2^i^	0.93	2.60	3.467 (2)	156

Symmetry code: (i) 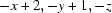.

**Fig. 1.**
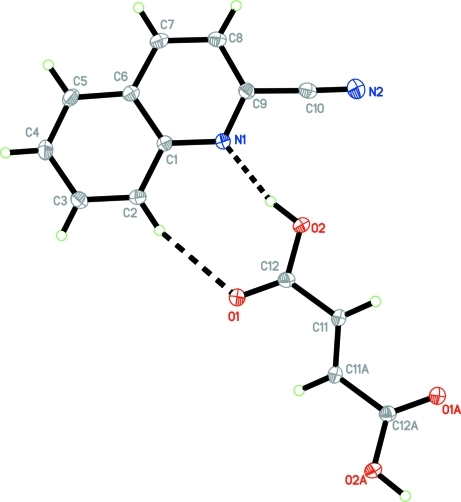
The molecular structure of the title compound showing 50% probability displacement ellipsoids for non-H atoms and the atom-numbering scheme. Atoms with suffix A were generated by the symmetry code -x, -y + 1, -z + 1. Hydrogen atoms are shown as spheres of arbitrary radius.

**Fig. 2.**
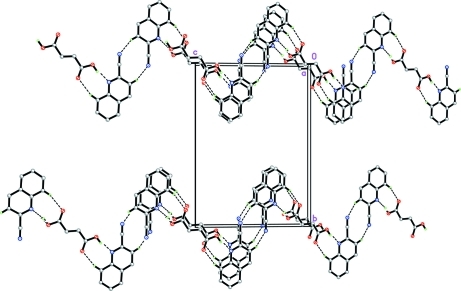
The crystal structure of the title compound, viewed along the a axis, showing the zigzag chains running along the c axis. Dashed lines indicate hydrogen bonds. H atoms not involved in the hydrogen bond interactions have been omitted for clarity.

## supplementary crystallographic information

### Comment

Heterocyclic molecules containing the cyano group are useful as drug intermediates. Syntheses of quinoline derivatives have been discussed earlier (Sasaki *et al.*, 1998; Reux *et al.*, 2009). In continuation of our previous work, we have synthesized a number of quinoline compounds to investigate the hydrogen bonding patterns in these compounds (Loh, Fun *et al.*, 2010; Loh, Quah *et al.*, 2010; Quah *et al.*, 2010). Here we report the synthesis of quinoline-2-carbonitrile fumaric acid.

The asymmetric unit of the title compound (Fig. 1) consists of one quinoline-2-carbonitrile molecule and a half-molecule of fumaric acid. The fumaric acid (C11/C12/O1/O2/C11A/C12A/O1A/O2A) lies on the inversion center generated by the symmetry code -*x*, -*y* + 1, -*z* + 1. The quinoline-2-carbonitrile (C1–C10/N1/N2) is almost planar, with an r.m.s. deviation of 0.008 (1) Å. The acid and base are linked together *via* pairs of intermolecular C2—H2A···O1 and O2—H1O2···N1 hydrogen bonds (Table 1), forming *R*_2_^2^(8) ring motifs (Bernstein *et al.*, 1995). The bond lengths (Allen *et al.*, 1987) and angles in the title compound are within normal ranges and comparable to those in the structure of quinoline-2-carbonitrile (Loh, Quah *et al.*, 2010).

In the crystal packing (Fig. 2), the carbonitrile molecules are further linked by intermolecular C8—H8A···N2 hydrogen bonds (Table 1), generating *R*_2_^2^(10) ring motifs (Bernstein *et al.*, 1995), and resulting in zigzag chains running along the *c* axis.

### Experimental

A hot methanol solution (20 ml) of quinoline-2-carbonitrile (39 mg, Aldrich) and fumaric acid (29 mg, Aldrich) were mixed and warmed over a magnetic stirrer hotplate for a few minutes. The resulting solution was allowed to cool slowly to room temperature. Colourless crystals suitable for X-ray diffraction appeared after a few days.

### Refinement

H1O2 was located from a difference Fourier map and refined freely (O—H = 0.92 (2) Å). The remaining H atoms were positioned geometrically with C—H = 0.93 Å and were refined using a riding model, with *U*_iso_(H) = 1.2 *U*_eq_(C).

### Crystal data

**Table:**

C_10_H_6_N_2_·0.5C_4_H_4_O_4_	*F*(000) = 440
*M**_r_* = 212.20	*D*_x_ = 1.448 Mg m^−^^3^
Monoclinic, *P*2_1_/*c*	Mo *K*α radiation, λ = 0.71073 Å
Hall symbol: -P 2ybc	Cell parameters from 3503 reflections
*a* = 3.7239 (1) Å	θ = 2.6–30.1°
*b* = 19.1958 (3) Å	µ = 0.10 mm^−^^1^
*c* = 13.6454 (2) Å	*T* = 100 K
β = 93.805 (1)°	Block, colourless
*V* = 973.27 (3) Å^3^	0.17 × 0.15 × 0.09 mm
*Z* = 4	

### Data collection

**Table:**

Bruker SMART APEXII CCD area-detector diffractometer	2566 independent reflections
Radiation source: fine-focus sealed tube	1983 reflections with *I* > 2σ(*I*)
graphite	*R*_int_ = 0.032
φ and ω scans	θ_max_ = 29.0°, θ_min_ = 1.8°
Absorption correction: multi-scan (*SADABS*; Bruker, 2009)	*h* = −5→5
*T*_min_ = 0.983, *T*_max_ = 0.991	*k* = −26→21
10682 measured reflections	*l* = −18→18

### Refinement

**Table:**

Refinement on *F*^2^	Primary atom site location: structure-invariant direct methods
Least-squares matrix: full	Secondary atom site location: difference Fourier map
*R*[*F*^2^ > 2σ(*F*^2^)] = 0.047	Hydrogen site location: inferred from neighbouring sites
*wR*(*F*^2^) = 0.128	H atoms treated by a mixture of independent and constrained refinement
*S* = 1.06	*w* = 1/[σ^2^(*F*_o_^2^) + (0.0637*P*)^2^ + 0.2928*P*] where *P* = (*F*_o_^2^ + 2*F*_c_^2^)/3
2566 reflections	(Δ/σ)_max_ < 0.001
149 parameters	Δρ_max_ = 0.37 e Å^−^^3^
0 restraints	Δρ_min_ = −0.26 e Å^−^^3^

### Special details

**Table:**

Experimental. The crystal was placed in the cold stream of an Oxford Cryosystems Cobra open-flow nitrogen cryostat (Cosier & Glazer, 1986) operating at 100.0 (1) K.
Geometry. All e.s.d.'s (except the e.s.d. in the dihedral angle between two l.s. planes) are estimated using the full covariance matrix. The cell e.s.d.'s are taken into account individually in the estimation of e.s.d.'s in distances, angles and torsion angles; correlations between e.s.d.'s in cell parameters are only used when they are defined by crystal symmetry. An approximate (isotropic) treatment of cell e.s.d.'s is used for estimating e.s.d.'s involving l.s. planes.
Refinement. Refinement of *F*^2^ against ALL reflections. The weighted *R*-factor *wR* and goodness of fit *S* are based on *F*^2^, conventional *R*-factors *R* are based on *F*, with *F* set to zero for negative *F*^2^. The threshold expression of *F*^2^ > σ(*F*^2^) is used only for calculating *R*-factors(gt) *etc*. and is not relevant to the choice of reflections for refinement. *R*-factors based on *F*^2^ are statistically about twice as large as those based on *F*, and *R*- factors based on ALL data will be even larger.

### Fractional atomic coordinates and isotropic or equivalent isotropic displacement parameters (Å^2^)

**Table:**

	*x*	*y*	*z*	*U*_iso_*/*U*_eq_	
O1	0.1218 (3)	0.38047 (6)	0.42628 (8)	0.0239 (3)	
O2	0.3112 (3)	0.45849 (6)	0.31757 (7)	0.0191 (3)	
C11	0.0375 (4)	0.50196 (8)	0.45325 (10)	0.0161 (3)	
H11A	0.0141	0.5445	0.4209	0.019*	
C12	0.1585 (4)	0.43988 (8)	0.39883 (10)	0.0153 (3)	
H1O2	0.393 (6)	0.4215 (12)	0.2830 (15)	0.042 (6)*	
C7	0.7889 (4)	0.28524 (8)	0.03215 (10)	0.0168 (3)	
H7A	0.8593	0.2596	−0.0211	0.020*	
C8	0.8084 (4)	0.35628 (8)	0.03082 (10)	0.0170 (3)	
H8A	0.8902	0.3798	−0.0229	0.020*	
C9	0.7002 (4)	0.39284 (8)	0.11370 (10)	0.0155 (3)	
C10	0.7209 (4)	0.46837 (8)	0.11449 (10)	0.0179 (3)	
N1	0.5786 (3)	0.36333 (6)	0.19310 (8)	0.0146 (3)	
N2	0.7411 (4)	0.52815 (7)	0.11298 (10)	0.0252 (3)	
C1	0.5589 (4)	0.29220 (8)	0.19492 (10)	0.0148 (3)	
C2	0.4324 (4)	0.25919 (8)	0.27928 (10)	0.0162 (3)	
H2A	0.3641	0.2857	0.3319	0.019*	
C3	0.4121 (4)	0.18825 (8)	0.28244 (11)	0.0179 (3)	
H3A	0.3308	0.1668	0.3379	0.021*	
C4	0.5123 (4)	0.14668 (8)	0.20288 (11)	0.0191 (3)	
H4A	0.4970	0.0984	0.2067	0.023*	
C5	0.6316 (4)	0.17730 (8)	0.12047 (11)	0.0183 (3)	
H5A	0.6933	0.1498	0.0680	0.022*	
C6	0.6612 (4)	0.25059 (8)	0.11479 (10)	0.0155 (3)	

### Atomic displacement parameters (Å^2^)

**Table:**

	*U*^11^	*U*^22^	*U*^33^	*U*^12^	*U*^13^	*U*^23^
O1	0.0376 (7)	0.0139 (6)	0.0214 (5)	0.0008 (5)	0.0117 (5)	0.0000 (4)
O2	0.0273 (6)	0.0154 (6)	0.0155 (5)	0.0003 (5)	0.0075 (4)	−0.0023 (4)
C11	0.0202 (7)	0.0116 (7)	0.0168 (7)	0.0001 (6)	0.0033 (6)	−0.0014 (5)
C12	0.0171 (7)	0.0159 (7)	0.0130 (6)	−0.0005 (6)	0.0013 (5)	−0.0005 (5)
C7	0.0169 (7)	0.0205 (8)	0.0130 (6)	0.0027 (6)	0.0013 (5)	−0.0023 (5)
C8	0.0173 (7)	0.0201 (8)	0.0139 (6)	0.0006 (6)	0.0032 (5)	0.0012 (5)
C9	0.0159 (7)	0.0156 (8)	0.0149 (6)	0.0004 (6)	0.0010 (5)	0.0010 (5)
C10	0.0193 (7)	0.0200 (8)	0.0145 (6)	−0.0002 (6)	0.0029 (5)	0.0015 (6)
N1	0.0172 (6)	0.0128 (6)	0.0139 (6)	0.0006 (5)	0.0016 (5)	0.0005 (5)
N2	0.0335 (8)	0.0183 (7)	0.0245 (7)	−0.0010 (6)	0.0062 (6)	0.0020 (6)
C1	0.0167 (7)	0.0143 (7)	0.0136 (6)	0.0010 (6)	0.0017 (5)	−0.0002 (5)
C2	0.0195 (7)	0.0165 (8)	0.0129 (6)	0.0008 (6)	0.0024 (5)	−0.0004 (5)
C3	0.0198 (7)	0.0173 (8)	0.0166 (7)	−0.0007 (6)	0.0017 (6)	0.0024 (6)
C4	0.0221 (8)	0.0126 (7)	0.0223 (7)	−0.0003 (6)	0.0002 (6)	−0.0005 (6)
C5	0.0219 (7)	0.0162 (8)	0.0170 (7)	0.0017 (6)	0.0017 (6)	−0.0043 (6)
C6	0.0159 (7)	0.0163 (8)	0.0142 (6)	0.0010 (6)	0.0010 (5)	−0.0015 (5)

### Geometric parameters (Å, °)

**Table:**

O1—C12	1.2108 (18)	C10—N2	1.150 (2)
O2—C12	1.3281 (16)	N1—C1	1.3676 (19)
O2—H1O2	0.92 (2)	C1—C2	1.4214 (19)
C11—C11^i^	1.326 (3)	C1—C6	1.4262 (19)
C11—C12	1.489 (2)	C2—C3	1.365 (2)
C11—H11A	0.9300	C2—H2A	0.9300
C7—C8	1.366 (2)	C3—C4	1.417 (2)
C7—C6	1.4181 (19)	C3—H3A	0.9300
C7—H7A	0.9300	C4—C5	1.369 (2)
C8—C9	1.4121 (19)	C4—H4A	0.9300
C8—H8A	0.9300	C5—C6	1.414 (2)
C9—N1	1.3287 (17)	C5—H5A	0.9300
C9—C10	1.452 (2)		
			
C12—O2—H1O2	113.4 (14)	N1—C1—C2	118.74 (12)
C11^i^—C11—C12	121.62 (18)	N1—C1—C6	121.86 (12)
C11^i^—C11—H11A	119.2	C2—C1—C6	119.40 (13)
C12—C11—H11A	119.2	C3—C2—C1	119.51 (13)
O1—C12—O2	125.09 (13)	C3—C2—H2A	120.2
O1—C12—C11	123.72 (13)	C1—C2—H2A	120.2
O2—C12—C11	111.19 (12)	C2—C3—C4	121.31 (13)
C8—C7—C6	119.98 (13)	C2—C3—H3A	119.3
C8—C7—H7A	120.0	C4—C3—H3A	119.3
C6—C7—H7A	120.0	C5—C4—C3	120.25 (14)
C7—C8—C9	117.87 (13)	C5—C4—H4A	119.9
C7—C8—H8A	121.1	C3—C4—H4A	119.9
C9—C8—H8A	121.1	C4—C5—C6	120.21 (13)
N1—C9—C8	124.88 (14)	C4—C5—H5A	119.9
N1—C9—C10	116.13 (12)	C6—C5—H5A	119.9
C8—C9—C10	118.98 (12)	C5—C6—C7	122.80 (13)
N2—C10—C9	178.33 (15)	C5—C6—C1	119.31 (12)
C9—N1—C1	117.52 (12)	C7—C6—C1	117.89 (13)
			
C11^i^—C11—C12—O1	17.0 (3)	C1—C2—C3—C4	−0.4 (2)
C11^i^—C11—C12—O2	−162.72 (18)	C2—C3—C4—C5	−0.2 (2)
C6—C7—C8—C9	0.3 (2)	C3—C4—C5—C6	1.0 (2)
C7—C8—C9—N1	−0.4 (2)	C4—C5—C6—C7	178.87 (14)
C7—C8—C9—C10	179.54 (14)	C4—C5—C6—C1	−1.2 (2)
C8—C9—N1—C1	0.5 (2)	C8—C7—C6—C5	179.47 (14)
C10—C9—N1—C1	−179.45 (13)	C8—C7—C6—C1	−0.4 (2)
C9—N1—C1—C2	179.50 (13)	N1—C1—C6—C5	−179.37 (14)
C9—N1—C1—C6	−0.5 (2)	C2—C1—C6—C5	0.6 (2)
N1—C1—C2—C3	−179.81 (14)	N1—C1—C6—C7	0.5 (2)
C6—C1—C2—C3	0.2 (2)	C2—C1—C6—C7	−179.50 (13)

Symmetry codes: (i) −*x*, −*y*+1, −*z*+1.

### Hydrogen-bond geometry (Å, °)

**Table:**

*D*—H···*A*	*D*—H	H···*A*	*D*···*A*	*D*—H···*A*
O2—H1O2···N1	0.92 (2)	1.83 (2)	2.7272 (16)	167 (2)
C2—H2A···O1	0.93	2.44	3.3300 (19)	161.
C8—H8A···N2^ii^	0.93	2.60	3.467 (2)	156.

Symmetry codes: (ii) −*x*+2, −*y*+1, −*z*.
